# S15-3: Defining Health Promoting Sports coaches: a systematic review

**DOI:** 10.1093/eurpub/ckae114.267

**Published:** 2024-09-26

**Authors:** Catherine O'Sullivan

**Affiliations:** World Athletics, Sweden

## Abstract

**Purpose:**

Coaches play a key role on the physical, mental, and social well-being of sports participants, and can foster health through sport’s informal educational nature. Nevertheless, sports coaches feel unequipped in regard to health promotion (HP). The objectives of this systematic encompass the identification of coaches' HP skills, as well as barriers and leverages in their acquisition.

**Methods:**

This systematic review is based on PRISMA-P guidelines. The search was run on 6 databases with following keywords: ((“sport* coach*” OR “sport trainer*” OR “sport* volunteer*”) AND (“skill*” OR “training” OR “behavior” OR “behaviour” OR “education”)) AND (“health”). Double blind analyze was used for selection and exclusion criteria were: not focused on grassroots sport coaches’ skills regarding health; no original English peer-review research article.

**Results:**

On the 2485 studies screened, 56 were included. Ten key skills of coaches in HP empowerment including athletes’ management, support health, education, appraisal, motivation, cooperation, prevention, communication, role model and self-management were identified. Numerous barriers, notably related to perceived constraints within their role, the pressure to sustain athletes' engagement in the activity, and their conceptualization of sport, leading to a concentration on performance-oriented practices rather than holistic sport development, such as HP were reported in the analysed studies. However, certain leverages, such as psychological factors, educational background, experiential learning, knowledge base, and adherence to guidelines have been found to support skills acquisition.

**Conclusions:**

Coaches skillset enables them to engage in athletes' health through motivational strategies, climate management, health support, and self-management techniques. Sports organization should therefore consider coaches as a health multiplier based on their support in regard to these skills acquisition.

**Funding:**

A partnership between Santé Publique France, Université de Lorraine and Université Côte d’Azur allowed and supported this work and by the Région Grand Est and Pole Biologie Medecine Santé of the Université de Lorraine.

